# Field Monitoring of Aflatoxins in Feed and Milk of High-Yielding Dairy Cows under Two Feeding Systems

**DOI:** 10.3390/toxins13030201

**Published:** 2021-03-11

**Authors:** Noemi Bervis, Susana Lorán, Teresa Juan, Juan José Carramiñana, Antonio Herrera, Agustín Ariño, Marta Herrera

**Affiliations:** 1 Instituto Agroalimentario de Aragón—IA2 (Universidad de Zaragoza-CITA), Facultad de Veterinaria, 50013 Zaragoza, Spain; bervis019@hotmail.com (N.B.); sloran@unizar.es (S.L.); tjuan@cita-aragon.es (T.J.); carramin@unizar.es (J.J.C.); aherrera@unizar.es (A.H.); herremar@unizar.es (M.H.); 2 Centro de Investigación y Tecnología Agroalimentaria de Aragón (CITA), 50059 Zaragoza, Spain

**Keywords:** aflatoxin B1, aflatoxin M1, feed, milk, cow, transfer ratio

## Abstract

Aflatoxin M1 (AFM1) is a hydroxylated metabolite of aflatoxin B1 (AFB1) that can be excreted in milk of cows after consuming contaminated feed. The aim of this study consisted of a field monitoring to assess the contamination levels of AFB1 in 60 feed samples from two feeding systems for high-yielding dairy cows and of AFM1 in the corresponding raw milk samples. The aflatoxins were analyzed by in-house validated methods based on high-performance liquid chromatography (HPLC) with fluorescence detection. AFB1 was detected in 55% of feed samples (mean 0.61 μg/kg, with 2 samples exceeding the European Union (EU) maximum level set at 5 μg/kg), with greater incidence and concentration in compound feed than in unifeed rations (*p* < 0.05). AFM1 was detected in 38.3% milk samples (mean 12.6 ng/kg, with 5 samples exceeding the EU maximum level set at 50 ng/kg), with a higher occurrence in milk of cows fed compound feed, as well as in spring milk compared to that produced in winter. The overall transfer ratio of aflatoxins from feed to milk was 3.22%, being higher in cows fed with compound feed and in spring milkings. In a selection of positive matched samples (*n* = 22), the ratio AFM1/AFB1 exceeded the European Food Safety Authority (EFSA) estimated 6% threshold for high-yielding dairy cows.

## 1. Introduction

The mycotoxins of greatest concern in food and feed safety are produced by various species of filamentous fungi belonging primarily to the genera *Aspergillus*, *Fusarium* and *Penicillium*. These fungi colonize a wide variety of agricultural products and can adapt to a wide range of environmental conditions. Because climate can profoundly affect the growth and distribution of molds and the production of mycotoxins, current climate change has the potential to increase the risks they pose to human and animal health [1].

Aflatoxins (AF) are genotoxic and carcinogenic agents, which include four main structural analogs, named AFB1, AFB2, AFG1, and AFG2. Animals fed products contaminated by aflatoxin B1 can transform it into aflatoxin M1 that is excreted in milk [2], posing a significant risk to public health [3]. The International Agency for Research on Cancer (IARC) has classified aflatoxins (B1, B2, G1, G2, M1) as Group 1, carcinogenic to humans [4]; they are related to liver cancer [5].

Aflatoxins are produced in agricultural crops by various *Aspergillus* species, but the two species of greatest concern are *A. flavus* and *A. parasiticus* [6]. Aflatoxin contamination in food and feed is most severe in tropical and subtropical areas around the world, where temperature and humidity conditions are optimal for the growth of aflatoxigenic molds [7]. In addition to the risk posed by primary contamination of crops with toxigenic molds and aflatoxins, it must not be forgotten that poor agricultural, manufacturing, transportation and storage practices can lead to increased contamination rates in the post-harvest phase.

Until recently, the European perspective on aflatoxin contamination was much focused on imported feed and food commodities from third-world countries of tropical and subtropical climate areas. Of course, the EU member states have also dedicated many efforts to the control measures to manage the aflatoxin risk in internal productions. However, a change in the traditional areas of incidence of aflatoxins is expected due to the increase in average temperatures due to climate change, especially in southern Europe [8,9]. Not surprisingly, in the last 15 years, a series of hot and dry seasons in several European countries, including Italy, Romania, Serbia and Spain, have led to the detection of aflatoxins in corn used for animal feed, as well as in the milk of cattle fed with these contaminated products [10]. Therefore, the possible aflatoxin contamination of raw materials used in animal feed and food grown in the EU should be reviewed, particularly in light of potential changes in climate [11]. Although compliance with EU regulations is usually high, continuous monitoring is needed in order to avoid negative impacts of climate change on mycotoxin patterns resulting in food and feed safety problems.

Milk is a main food for the growth of children and young people, whose vulnerability is notable since they are more sensitive to toxic effects than adults [12]. Therefore, the appearance of aflatoxin M1 in cow’s milk and dairy products is a serious food safety problem [13]. The aflatoxin transfer rate from feed (AFB1) to milk (AFM1) depends on the level of contamination, the amount ingested, the duration of exposure, the type of feeding and the cow’s metabolism, among other factors [14]. The EFSA has estimated that the aflatoxin transfer rate from cattle rations to milk is 1–2% on average, although in high-productivity cattle it can increase to 6% [7].

High-yielding dairy cows, which dominate today’s farms, could represent a category of animals in which a higher rate of transfer of aflatoxin M1 to milk could result in concentrations exceeding the current EU limits for raw milk. Therefore, current data on the rate of aflatoxin transfer to milk should be generated taking into account modern production systems, high-yielding animals, and different dairy cow feeding management systems. In Spain, two general types of feeding systems predominate in intensive dairy farms: confinement systems with totally mixed rations (TMRs or unifeed) and confinement systems in which compound feed concentrates and forages are used to complete the ration. The increase of average temperatures and precipitation variations may affect the incidence and patterns of aflatoxin contamination in many of the ingredients used for dairy cow feed, such as maize, oilseeds, silages and preserved grass, which can be used in varying proportions in both feeding systems. In addition, the quantity and quality of feed will be affected by climate change [15].

In the European Union (EU) maximum levels have been set for aflatoxin B1 and the total sum of aflatoxins (B1, B2, G1, G2) in food products, and aflatoxin M1 in milk by Regulation (EC) No. 1881/2006 [16]. At the same time, in products intended for animal feed, maximum limits for aflatoxin B1 have been established by Directive 2002/32/EC [17]. For comparison, the maximum aflatoxin M1 content in milk is 0.05 μg/kg, while the maximum aflatoxin B1 content in compound feed for dairy cows is 5 μg/kg.

The aim of this study consists of a field monitoring to examine the contamination levels of AFB1 in two types of complete feed intended for high-yielding dairy cattle (unifeed rations vs. compound feed) during two seasons (spring and winter). At the same time, the levels of contamination by aflatoxin M1 in the corresponding raw cow’s milk samples were studied to evaluate the aflatoxin transfer rates from feed to milk.

## 2. Results and Discussion

### 2.1. Aflatoxin B1 in Feed

From December 2015 to June 2016, a total of 60 different complete feed samples for dairy cattle were analyzed in a field monitoring study. The feed samples belonged to two feeding systems: 38 samples from Total Mixed Rations (TMR unifeed) and 22 samples from compound feed and forages. Table 1 shows the results obtained in the determination of AFB1 in the samples, classified by type of feeding system and by sampling season (spring: *n* = 35; winter: *n* = 25). The global prevalence of aflatoxin B1 was 55% (33 out of 60 samples), being higher in compound feed (19 positive samples, 86%) than in unifeed rations (14 positive samples, 36.8%). Regarding the sampling season, the incidence of aflatoxin B1 in spring feed samples (57.1%) was similar to that observed in winter feed samples (52%), so no seasonal effect was observed.

The mean ± standard deviation (SD) concentration of aflatoxin B1 in the 60 samples of cattle feed was 0.61 ± 1.14 μg/kg, in a range of 0.07 to 5.17 μg/kg. The concentration of AFB1 was significantly higher in compound feed (0.99 ± 1.56 μg/kg) than in unifeed rations (0.39 ± 0.75 μg/kg) (*p* < 0.05). Likewise, the mean concentration of aflatoxin B1 in winter feed samples (0.73 ± 1.43 μg/kg), was higher than that found in the spring ones (0.53 ± 0.89 μg/kg), although this difference was not statistically confirmed (*p* > 0.05).

The variation observed in aflatoxin contamination between the 60 different feed samples was because they came from different sampling areas (18 farms from four provinces of two regions) and different sampling days during the sampling period (December 2015 to June 2016), and pertain to different feeding systems (unifeed and concentrates). Regarding ingredients, the main differences in contamination levels between samples could be due to the presence of susceptible ingredients such as cottonseed. However, the differences in aflatoxin B1 levels between sampling years (2015 vs. 2016) and among the four provinces in which the farms were located were not significant (data not shown).

Of the total of 60 different feed samples analyzed in our study, only 2 samples (3.3%) exceeded the maximum aflatoxin B1 level in compound feeds for dairy cows, established at 5 μg/kg. In both cases, they were compound feeds with aflatoxin B1 concentrations of 5.10 and 5.17 μg/kg, respectively, and coincided with samplings carried out in winter. Despite the fact that these samples exceeded the maximum content established for AFB1, the cows that consumed them produced milk that did not exceed the maximum content of aflatoxin M1 established at 50 ng/kg (27.5 and 42.1 ng/kg, respectively).

The incidence of aflatoxin B1-contaminated feed samples found in this study can be considered high, since it reached 55% of the samples. Our incidence data agree with the range reported for raw materials in annual Biomin’s worldwide mycotoxin surveys [18]. By comparison, the incidence rates of aflatoxins in raw materials from southern Europe were 55% of the samples in 2014 and 16% in 2018, indicating a large inter-annual variation subject to climatic changes. In addition, in a ten-year survey of the global mycotoxin occurrence in feed (samples collected from 100 countries from 2008 to 2017), the global incidence of aflatoxin B1 was 23%, while in southern Europe was 28.9% [19].

The higher concentration of aflatoxin B1 in compound feed as compared to unifeed may be due to some of the commodities used in its manufacture that are very susceptible to aflatoxins, such as cottonseed [20,21]. The compound feed investigated in the present study contained 4.5% of cottonseed, a known source of aflatoxins in feed [22], that was absent in the unifeed rations. In the review by van der Fels-Klerx et al. [23] on ingredients used in animal feed, several risk categories were identified in relation to aflatoxins, assigning the highest risk to peanuts, corn and oilseeds. In Spain, peanuts are practically not used for the manufacture of compound feed, but both corn and its derivatives, as well as cottonseed, are common ingredients [24].

Therefore, it is not surprising that our compound feed samples were more contaminated by aflatoxin B1 due to their formulation with susceptible ingredients. Our results coincide with an investigation carried out on the rates of AFB1 in rations for dairy cattle (*n* = 160) [25], in which the incidence was 50% and the concentrated products were more contaminated (7.67 ± 0.80 μg/kg) than the forages (0.41 ± 0.14 μg/kg).

Within the efforts to control mycotoxin contamination in animal feed, several studies have been carried out to evaluate the incidence of mycotoxin contamination in feed and feed raw materials. In Navarra (Spain), Hernández and Navarro [26] analyzed the aflatoxin B1 incidence in 78 samples of dairy cow rations from 40 farms. Although the incidence was very high (74% positivity), the contamination rates were an order of magnitude lower than those found in our study and none of the samples exceeded the established maximum content. In another study carried out in Spain, about one-third (34.7%) of the total mixed rations for cows were positive for total AFs in a range of 0.05–6.45 μg/kg, and 12.4% were positive for AFB1 [21].

The levels of aflatoxin B1 in our study are in line with the values reported in the annual mycotoxin surveys of products intended for animal feed, carried out by the animal feed company Biomin [18]. These surveys show year-on-year variability mainly due to weather-related factors. In the 2015 survey, the average AFB1 concentration in feedstuff from Europe was 6 μg/kg with a maximum of 153 μg/kg. In the 2016 Biomin survey, the mean concentration was 4 μg/kg with a maximum of 18 μg/kg. These levels are in line with those reported for European feedstuff in another study [19]. However, there are reports from other areas showing higher levels of AFB1 in feed, such as Tunisia [27], Ethiopia [28] and China [29].

In Europe, with regard to animal feed, a low level of mycotoxin contamination is found in more than 50% of the samples, although it is not frequent to find samples with concentrations above the established maximum limits [30]. Rodrigues and Naehrer [31] studied the prevalence of aflatoxins in 199 European samples, with 16.6% positivity and a maximum value of 103 μg/kg. Griessler et al. [32] analyzed 127 samples from southern Europe, finding a positivity for aflatoxins of 25%, with a maximum concentration of 66 μg/kg.

In general, warm climates are known to be favorable for the growth of aflatoxigenic molds; however, not all studies agree regarding the seasonal influence on aflatoxin rates in animal feed. Thus, Hernández and Navarro [26] analyzed aflatoxin B1 levels in 78 samples of dairy cow rations in Navarra (Spain) and pointed to spring as the season with the highest level of aflatoxin B1 in the feed (0.086 μg/kg), followed by winter (0.075 μg/kg), summer (0.030 μg/kg) and autumn (0.017 μg/kg). These results differ from ours, in which no significant seasonal differences were found.

Mycotoxin incidence patterns are changing as a consequence of the increase in average temperatures due to climate change, which especially affects southern European countries [33,34,35]. Binder et al. [36] reported maximum levels of AFB1 in samples destined for animal feed from northern Europe (60 μg/kg), central Europe (311 μg/kg), and southern Europe (656 μg/kg), indicating an association between climate and the presence of aflatoxin B1. Likewise, Italian researchers detected AFB1 in cattle feed and AFM1 in cow’s milk above the maximum allowed content in the EU, in 8.1% and 1.7% of samples, respectively, which they associated with one year with especially warm climate [37].

In Serbia during 2013, 281 samples of compound dairy cow feed mixes were examined, 67 samples (23.8%) contained AFB1 above the maximum limit. The commodity that caused the contamination was the corn kernel used as a flour concentrate. High frequencies of aflatoxins have also been published in maize for animal consumption in Croatia, when 38% of the 633 maize samples analyzed were contaminated with aflatoxins, with a very high mean value of 81 μg/kg [38].

### 2.2. Aflatoxin M1 in Milk

In parallel to the mycotoxin analysis in feed, the presence and concentration of aflatoxin M1 were analyzed in the 60 samples of raw cow’s milk, which corresponded to the analyzed samples of dairy cattle feed in this field monitoring study. Table 2 shows the results obtained in the determination of AFM1 in bulk milk samples, ordered by type of livestock feed (unifeed rations: *n* = 38; compound feed: *n* = 22) and season of the year in which the milking was performed (spring: *n* = 35; winter: *n* = 25). The global incidence of aflatoxin M1 was 38.3% (23 of 60 samples), being higher in milk from cows fed compound feed (12 positive samples, 54.5%) than in those fed unifeed rations (11 positive samples, 28.9%). Regarding the sampling season, the incidence of aflatoxin M1 in spring samples (40%) was similar to that observed in winter samples (36%).

The mean concentration ± standard deviation (SD) of aflatoxin M1 in the 60 milk samples was 12.6 ± 19.1 ng/kg, being higher again in milk from cows fed compound feed (18.8 ± 21.8 ng/kg) than in those fed unifeed rations (9.00 ± 16.6 ng/kg), although this difference was not statistically confirmed (*p* > 0.05). As indicated above, the compound feed contained cottonseed, which was absent in the unifeed rations, as well as other oilseeds such as rapeseed. A trend was observed in which aflatoxin M1 contamination was higher in spring milkings (15.3 ± 22.3 ng/kg) than in winter ones (8.8 ± 12.8 ng/kg), although it has not been statistically confirmed (*p* > 0.05).

Of the total of 60 different milk samples, 5 samples (8.3%) exceeded the maximum content of aflatoxin M1 in raw milk, established at 50 ng/kg. Three of these samples, with AFM1 concentrations of 67.2, 66.1 and 50.2 ng/kg, corresponded to milk from cows fed with compound feed, while the other ones (58.1 and 54.7 ng/kg) came from cows fed unifeed rations. It should be noted that all these milk samples whose levels of aflatoxin M1 exceeded the established maximum content coincided with spring samplings.

Many studies found aflatoxin M1 in cow milk, although only a small proportion of the contaminated samples exceeded the maximum tolerable EU limit of 0.05 μg/kg. There are several studies available in Spain on aflatoxin M1 contamination in milk. Rodríguez et al. [39] analyzed 92 samples of raw cow’s milk collected in dairy farms in the province of León. The positivity to AFM1 was 3.3% (3 samples) and concentrations were in the range of 10 to 50 ng/kg, so no sample exceeded the maximum content allowed by European Union legislation. In Navarra, Gómez-Arranz [40] analyzed the aflatoxin M1 levels in 477 samples of cow’s milk over a year. The contamination rates were similar to those found in our study. Likewise, the seasonal effect also indicated spring as the season with the highest average level of aflatoxin M1 in milk (11 ng/kg), followed by winter (7 ng/kg) and summer (3 ng/kg); aflatoxin M1 was not detected in the fall samples.

In Catalonia, Cano-Sancho et al. [41] detected aflatoxin M1 in 68 of 72 ultra-high temperature (UHT) milk samples (94.4%), with a maximum value of 14 ng/kg. The high incidence of AFM1 is a cause for concern, as milk is a recommended dietary staple for all ages, especially children and adolescents [42]. In another study, AFM1 was detected in 18.9% of bulk milk samples, with concentrations ranging from 0.009 to 1.36 μg/kg [21].

Since 2013, Andalusia (southern Spain) has incorporated the control of the presence of aflatoxin M1 in milk throughout the production phases of the dairy sector, through control programs developed by the competent authorities. Some data point to an increase in the incidence of aflatoxin M1 in milk, especially in hot and dry years when livestock rations may contain high levels of aflatoxin B1 [43]. In 2016, 56 positive cases for AFM1 were registered in milk, while in 2017 there were 16 cases reported [44].

The incidence of AFM1 has been reported in various locations around the world, and many of the studies expressed concern about AFM1 contamination levels above the maximum level set by European Union (EU) legislation (0.05 μg/kg), although AFM1 levels often do not exceed the maximum level set by the Food and Drug Administration (FDA) in the US (0.50 μg/kg) [45]. The data collected from studies carried out in recent years show that the incidence of AFM1 in milk samples is relatively low in European countries (lower than 20%), while studies in Asian countries show that the occurrence of AFM1 can reach 100% samples [46].

Flores-Flores et al. [47] have reviewed the presence of AFM1 in cow’s milk from various parts of the world. Of the 22,189 milk samples analyzed that were taken into account, at least 9.8% of them (2190 samples) exceeded the maximum AFM1 content established by the EU. Regarding the number of non-compliant samples per continent, 1709 came from Asia, 253 from Africa, 119 from Europe and 109 from America.

AFM1 contamination in milk and dairy products shows variations according to geographic region, environmental conditions, diversity of agricultural systems, availability of green forage, feed ingredients and consumption of feed concentrates, among others [46,48]. A seasonal variation in AFM1 contamination of milk has been previously reported, with an increase in mycotoxin during the winter period, but directly linked to the increase in concentrate feed in the animal’s diet [49]. This effect has been generally attributed to the fact that animals consume less concentrated food in summer because they also feed on green fodder and grass [48]. In China, Xiong et al. [50] evaluated the contamination of raw milk during different seasons and found that the presence of AFM1 was significantly higher during winter (123 ng/kg), compared to the remaining seasons, spring 29 ng/kg, summer 32 ng/kg and fall 32 ng/kg. In Croatia, Bilandžić et al. [51] also verified the distribution of AFM1 concentration in milk during the different seasons and found that the concentration was statistically higher between January and April (0.036 to 0.059 μg/kg) than between June and September (0.012 to 0.015 μg/kg).

Several studies agree that milk from grass-fed animals has lower levels of AFM1 compared to milk from animals fed compound feed and stored products. In the study by Çeçen [52], it was shown that grazing has an important effect on the decrease of AFM1 in milk. Thirty milk samples from farmhouse animals were investigated and compared to 31 samples from grazing animals. Only one sample of milk from grazing animals was found to have AFM1 (3.22%), while 23 samples from animals that were fed in stables (76.66%) were contaminated with AFM1. Therefore, the type of food consumed (concentrates, forages, pastures), may be behind the seasonal effect observed in the incidence and concentration of AFM1 in cow’s milk.

### 2.3. Transfer Ratio of Aflatoxins from Feed to Milk

From the analysis of our results, it is derived that the mean aflatoxin transfer ratio in all matched samples of feed and milk was 3.22% (Table 3), ranging from 0 to 36%. The mean transfer ratio was higher in the cows fed compound feed (5.07%) than in those fed by unifeed rations (2.15%), although this difference was not statistically confirmed (p > 0.05). Likewise, the effect of seasonality showed a higher transfer rate in spring milks (4.17%) compared to winter ones (1.89%), although the difference was again not significant (*p* > 0.05).

There were 22 matched samples in which aflatoxins were detected in both feed and the corresponding milk. However, there were 11 samples in which feed showed positive for AFB1 (ranging from 0.08 to 0.63 μg/kg dry matter), but AFM1 was not detected in the corresponding milk (transfer ratio was considered to be zero). Also, there was a milk sample positive to AFM1 (11.71 ng/kg) whose cows were fed a feed that was apparently free of aflatoxin B1 (lower than the limit of detection 0.03 μg/kg). A possible explanation for these discrepancies is that aflatoxins in feed and milk are indeed related, but the correlation between the two is not very strong due to different factors of variation such as the heterogeneous distribution of mycotoxins in raw materials and the sampling procedures. However, it is well accepted that the monitoring of feedstuffs is a useful tool in order to prevent and minimize aflatoxin-contamination of milk.

In a selection of positive matched samples in feed and milk (*n* = 22), the mean transfer rate was 8.79%, with a range of 0.76 to 36% (Table 4). In the two samples of cattle feed whose aflatoxin B1 level exceeded the maximum content of 5 μg/kg (#16 and 17), the transfer rate was 1.15% and 0.76%, respectively. In the five samples of raw milk whose aflatoxin M1 rate exceeded the maximum content of 50 ng/kg (#3, 8, 12, 14 and 15), the transfer rate ranged between 2.8 and 29.78%, with an average ratio of 15.22%.

There are studies that indicate that the various ingredients of the rations (cotton, corn, peanuts) lead to variations in the aflatoxin mixture that, eventually, will be transformed into AFM1 [53]. These authors calculated that the aflatoxin transfer rate to milk is higher when the rations contain contaminated cottonseed than when they contain contaminated corn. Therefore, the type of contaminated commodity used in the concentrate not only determines the composition of natural aflatoxins, but can also affect aflatoxin carry-over [54]. It has been reported that concentrated feeds and ingredients such as maize and cottonseed are highly correlated with the levels of AFM1 in milk [55,56].

Han et al. [57] observed that, although all the analyzed samples of feed rations for livestock were within the AFB1 levels allowed by the European Commission (5 μg/kg), the AFM1 content in 3 out of 200 milk samples was above the maximum levels established by the EU (50 ng/kg). Battacone et al. [58] also reported that there is no guarantee that the AFM1 concentration in milk will always be lower than the EU legal limit, even when the AFB1 levels in animal feed are within the limits.

The aflatoxin transfer rate from feed (AFB1) to milk (AFM1) depends on the level of contamination, the amount ingested, the duration of exposure, the type of feeding, the cow’s metabolism and its health status, among other factors [14,59]. Van Eijkeren et al. [53] proposed a stationary transfer model from AFB1 to AFM1. A fraction of the daily intake of AFB1 (μg/day) is absorbed through the intestine, depending on the source of contamination (corn, cotton, peanuts), the composition of the concentrate and the total composition of the compound ration. The absorbed amount of AFB1 determines the plasma concentration, which is partly eliminated by urinary excretion or by transformation into metabolites such as AFM1. Once AFM1 has formed in the liver, it circulates through plasma and some of it is excreted in milk.

EFSA has estimated that the transfer rate from aflatoxin B1 in feed to aflatoxin M1 in cow’s milk is 1–2% on average, although in high-productivity cattle it can increase to 6% [7]. However, this transfer rate may vary in individual animals, from day to day and from milking to milking, as it is influenced by the factors noted above. For high-performance dairy cows with a production of up to 40 L of milk per day, aflatoxin transfer percentages of up to 6.2% have been previously reported, especially in the first phase of lactation [60]. In contrast, other researchers have found transfer rates of only 0.56%, such as Xiong et al. [50], in an experiment with Holstein cows that produced 21.3 kg of milk per day. Britzi et al. [61] suggested that milk production is the main factor affecting the carry-over rate, with an average carry-over rate of 2.5% for low production cows (<35 L/day) and 5.4% for high-production cows (>35 L/day).

In Spain, the transfer ratio of AFB1 from total mixed rations to AFM1 in raw cow’s milk was reported to be between 0.6 and 6% [21], a range comparable to that found in our study. Likewise, other studies carried out in Serbia indicate that between 0.3 and 6.2% of AFB1 ingested by cows is transformed into AFM1, and the highest amount of AFM1 is present in milk between 6 and 24 h after cows have eaten the contaminated food. After that period, if the animal no longer ingests contaminated feed, the amount of AFM1 begins to decrease until complete elimination after 72 to 96 h [62].

In our study, the highest transfer rate occurred in cows fed compound feed (5.07%), which is in line with the 6% value indicated by the EFSA for high-productivity cows. This transfer rate is much higher than the value of 0.54% reported for Holstein cows by Galvano et al. [63]. However, the cows used in this study were in the last stage of lactation, when the transfer rate was markedly reduced.

Other researchers [64] reported a 2.35% aflatoxin transfer in Friesian Holstein cows, similar to that of 3.22% found on average in our study. Likewise, Masoero et al. [65] in a similar study with Holstein cows, reported a transfer of 1.29% in animals with low milk production (21.2 kg/day) and of 2.70% in high-production cows (41.8 kg/day), in the same line as our results. Other authors such as Aslam et al. [59] observed a transfer of aflatoxins from feed to milk of 15%, very high compared to the rest of the authors.

## 3. Conclusions

Aflatoxin B1 and M1 were ubiquitously present in dairy cattle feed and raw cow´s milk samples (55% and 38.3%, respectively). Overall, the incidence of aflatoxin B1 in feed in the present field monitoring study indicated the need to apply integrated strategies for the prevention and reduction of aflatoxin contamination in products intended for animal feed, especially taking into account the predictions of increased risk of aflatoxins due to climate change. Likewise, the incidence data of aflatoxin M1 in milk indicated a public health risk that would justify an integrated plan for aflatoxin surveillance and control throughout the production chain.

The type of dairy cattle feed could have influenced the transfer ratio of aflatoxins from feed to milk as higher aflatoxin transfer rates were observed in dairy cows fed compound feed than in those fed unifeed rations, probably due to the presence of highly susceptible ingredients in the concentrates.

In summary, there is a need for planning an integrated surveillance, control and response system for the presence of aflatoxins throughout the entire food chain. The scope of the plan should cover the different stages of primary production that intervene in the raw milk production chain, that is, from agricultural production to the output of raw milk from livestock farms, passing through the feed industries; in the agricultural sector, through the implementation, by farmers and other producers of raw materials, of good agricultural practices for the prevention and control of aflatoxins in their crops, during harvest and in storage; in the animal feed sector, with the development of a program for the surveillance and control of aflatoxins in feed ingredients, based on the control of suppliers, the implementation of a traceability system and the performance of sampling and analysis; in the livestock sector, with the implementation of a series of specific measures for the surveillance and control of aflatoxins in dairy farms.

## 4. Materials and Methods

### 4.1. Description of Sampling Zones

All sampled dairy farms (*n* = 18) were situated in northern Spain (León and Burgos provinces in the Castilla-León region, and the Huesca and Zaragoza provinces in the Aragón region). These two regions represent high milk production areas in northern Spain. Sampling of feed and milk took place in winter season (December 2015, January and February 2016; 25 samples) and spring season (March to June 2016; 35 samples). According to the Köppen climate classification [66], León has Csb climate (temperate with dry or temperate summer), Burgos has Cfb climate (temperate with a dry season and temperate summer), Zaragoza has BSk climate (cold steppe), while Huesca has Cfa (temperate with a dry season and hot summer). The year 2015 was extremely warm in Spain, with an average temperature of 16.00 °C, a value that is 0.94 °C higher than normal (reference period 1981–2010), and it was very dry in the whole of Spain. The year 2016 was very warm in Spain, with an average temperature of 15.8 °C, and it was a wet year in the whole of Spain with an average rainfall around 682 mm, which is 5% more than the normal value according to the 1981–2010 reference period [67].

In the present field monitoring study of the incidence of aflatoxins in animal feed and raw cow’s milk, all the herds were high-yielding cows, Holstein-Friesian, housed in free stalls, with approximately 700 kg live weight. On average, each animal consumed 25 kg/day of dry matter (~50 kg fresh weight) and produced 40 kg of milk per day. The studied farms varied in the feeding system for the dairy cows. In one group of farms called “unifeed rations”, the complete feed was formulated as TMR (total mixed ration) using a unifeed system in the farm. In the other group called “compound feed”, the complete feed consisted of compound feed supplemented with forages.

### 4.2. Feed and Milk Samples

The “unifeed" rations (*n* = 38) were prepared daily in the same farms, from the usual raw materials for feeding dairy cows, with a predominance of maize and its derivatives (silage, flour, pastone). A typical unifeed formula in the studied farms included maize silage (50%), ray-grass silage (20%), maize flour (7%), maize pastone (5%), sugar beet molasses (5%), soy flour (5%), soybean meal (4%), mix of vitamins, minerals and other nutritional additives (4%). On average, the unifeed rations investigated in this study contained 52.5% forage dry matter and 47.5% concentrate dry matter.

On the other hand, the “compound” rations (*n* = 22) consisted of a compound feed for high-yielding cows (provided 45–50% of dry matter) supplemented with green and dry forages (provided 50–55% of dry matter). The typical compound feed formula in the studied farms contained maize (30%), soybean meal (18%), barley (13%), corn gluten (10%), palm extract flour (6%), bran from wheat (5%), sorghum (5%), mineral salts (5%), cottonseed (4.5%), rapeseed oil (2%) and molasses and beet pulp (1.5%). Supplementary forages included corn silage, ryegrass silage, alfalfa and cereal straw.

Both “unified” and “compound” complete feed samples were taken representatively from the mangers of the cows in production, mixing and sampling 2 kg in a nylon ziplock bag. During the day, four subsamples of 500 g were taken by specialized technicians taking into account the different particle size of the feed ingredients. Finally, the subsamples were aggregated and each farm provided a bulk sample of 2 kg in order to be representative of the whole. In addition, in order to try to minimize the sampling error, feed samples were dried and ground before being analyzed. None of the samples tested contained mycotoxin adsorbents.

The corresponding milk samples were taken within 24 h from the refrigeration tank of the milking parlor after agitation during 10 min to have the milk thoroughly mixed. Then, a clean and sanitized dipper was used in triplicate from the top of the tank and the representative sample was taken into 1 L plastic cans.

To account for seasonal differences, 35 samplings were done in spring while 25 were done in the winter. The farm personnel were in charge of taking the samples, guaranteeing traceability between each sample of cattle feed and the corresponding milk. For the sampling, we were also assisted by a national dairy company, the Veterinary Technical Cabinet (GTV) and the Center for Agrifood Research and Technology of Aragon (CITA).

All samples were identified and kept refrigerated at 4 °C until transfer to the laboratory. The samples of products destined for animal feeding were dried in an oven at 60 °C until a moisture value of 12%. Then, all samples were kept frozen at −20 °C until they were prepared for analysis. The preparation of feed samples consisted of grinding in a Mahlkönig EG-43 mill (Hamburg, Germany), to obtain samples with a homogeneous particle size.

### 4.3. Chemical and Reagents

Stock solutions of aflatoxin B1 and M1 were purchased from Sigma-Aldrich (St. Louis, MO, USA) and consisted of 2 μg/mL of AFB1 and 0.5 μg/mL of AFM1 in acetonitrile, respectively. Calibration curves for AFB1 and AFM1 were prepared at concentrations of 0.25, 0.5, 1, 2.5 and 5 ng/mL for AFB1 and 0.025, 0.050, 0.075, 0.100, 0.150 and 0.200 ng/mL for AFM1. Working calibration solutions were prepared with mobile phase, stored at 4 °C and renewed every week. The immunoaffinity columns (IAC) AflaTest WB SR and Afla M1 were acquired from VICAM (Watertown, MA, USA). Deionized water was obtained from a Millipore milli-Q water purification system (Mildford, MA, USA). HPLC-grade acetonitrile and methanol were supplied from Scharlau (Scharlab, Barcelona, Spain) and sodium chloride was purchased from Panreac (Barcelona, Spain). Certified reference materials from Biopure (Romer, Getzersdorf, Austria) were used for method validation: maize flour contaminated with AFB1 (QCM1C2, level 8.8 μg/kg ± 3.1 μg/kg) and milk powder contaminated with AFM1 (ERMBD 284, level 0.44 μg/kg ± 0.06 μg/kg).

### 4.4. HPLC Equipment and Chromatographic Conditions

The system consisted of an Agilent 1100 Series HPLC coupled to a fluorescence (FLD) detector (Agilent Technologies, Barcelona, Spain) with excitation and emission wavelength of 365 and 435 nm, respectively. Separation was carried out on a LC column Ace 5 C18, 250 × 4.6 mm, 5 μm particle size (Análisis Vínicos, Ciudad-Real, Spain). A manual injector system, equipped with a 100 μL injector loop and a 250 μL syringe, was used. The isocratic mobile phase for aflatoxin B1 was methanol/acetonitrile/water (40:10:50, *v*/*v*/*v*) and for aflatoxin M1 was acetonitrile/water (25/75, *v*/*v*), both pumped with a flow rate of 1.0 mL/min. The fluorescence intensity of AFB1 was improved with postcolumn derivatization (PHRED Photochemical reactor) (LCTech UVE, Dorfen, Germany) set at 254 nm.

### 4.5. Analytical Methods

Aflatoxin B1 in feed was analyzed by method EN 17375:2006 [68] with some modifications. Briefly, a 25 g sample was mixed with 2.5 g of sodium chloride and 50 mL of 80% methanol and then homogenized for 1 min. The mixture was filtered by Whatman No. 4 filter paper (Symta, Madrid, Spain), and 10 mL of the filtrate was mixed with 40 mL of milli-Q water. Then, 10 mL of the diluted filtrate was passed through immunoaffinity cleanup column at a flow rate of 1–2 drops per second. The column was then washed with milli-Q water and aflatoxin B1 was eluted with 1 mL of methanol. Later, 1 mL of milli-Q water was added to the eluate before filtering with 0.45 μm filter and injecting 100 μL into the LC-PHRED-FLD system.

Aflatoxin M1 in milk was analyzed by method EN 14501:2007 [69] with some modifications. Then, 100 mL of liquid milk samples were warmed to 37 °C in a water bath (Stuart Scientific, Staffordshire, UK) for ten minutes and then centrifuged at 4000 rpm (Hettich, Guipúzcoa, Spain) for 15 min to separate the fat layer. The extract (lower phase) was filtered through Whatman No. 4 filter paper. About 50 mL of the filtrate was transferred into a syringe barrel attached to an immunoaffinity column cleanup and passed at 1–2 drops per second. The column was rinsed with 10 mL of milli-Q water for impurities removal and later, 1.25 mL of acetonitrile/water (3:2 *v*/*v*) and 1.25 mL of milli-Q passed through the column to elute aflatoxin M1. The eluate was filtered with 0.45 μm filter and 100 μL were injected into LC-FLD system.

The analytical methods were validated in-house in terms of linearity, sensitivity, accuracy and precision. Linearity was assessed by constructing five-point calibration curves over the calibration range of 0.25 to 5 ng/mL for AFB1 and 0.025 to 0.200 ng/mL of AFM1. Linear regression lines were plotted using the peak area versus analyte concentrations and linearity was described by linear regression analysis and was expressed as coefficient of determination (R^2^) above 99%. The limits of detection (LOD) and quantification (LOQ) were determined for a signal/noise ratio of 3 and 10, respectively, using blank samples of feedstuff and milk. These blank samples were spiked at different AFB1 levels in feed (2.5, 5 and 10 μg/kg), and at different AFM1 levels in milk (0.025, 0.05 and 0.10 μg/kg). The precision was evaluated in terms of relative standard deviation (RSD%) from independent replicate analysis both intra-day (repeatability) and inter-day (reproducibility).

For AFB1 in feed, percent recovery ranged from 89.2 to 96.0%, repeatability (RSDr) was lower than 5%, reproducibility (RSDR) was lower than 10%, limit of detection (LOD) was 0.03 μg/kg and limit of quantification (LOQ) 0.1 μg/kg. For AFM1 in milk, percent recovery ranged from 96.6% to 98.6%, repeatability was <10%, reproducibility (RSDR) was lower than 15%, LOD was 8 ng/kg and LOQ 25 ng/kg. The triplicate analysis of the certified reference material QCM1C2 yielded a value of 8.36 ± 1.4 μg/kg of aflatoxin B1, which corresponded to a recovery value of 95%. The analysis of the certified reference material ERMBD 284 yielded a value of 0.43 ± 0.06 μg/kg of aflatoxin M1 (*n* = 3), which corresponded to a recovery value of 97.7%. All recovery values were within the performance criteria set by Commission Regulation (EC) No. 401/2016 [70] that establishes recovery values of 70–110% at AFB1 concentration levels between 1 and 10 μg. In the case of AFM1, the recoveries also fulfilled the requirements at different concentration levels: 60–120% in the range of 0.01–0.05 μg/kg and 70 to 110% at levels above 0.05 μg/kg. Likewise, the method was compliant in terms of RSDr% (repeatability) and RSDR% (reproducibility) [70]. Additionally, for analytical quality control, our laboratory participated in worldwide interlaboratory comparison rounds organized by Romer Labs during 2017 (Ref. CSSMY013-M17411A) and 2019 (CSSMY017-M19411AF), obtaining satisfactory results in terms of z-score.

### 4.6. Calculation of the Transfer Ratio of Aflatoxins from Feed to Milk

To calculate the transfer ratio of aflatoxins from feed (AFB1 ingested in μg/day) to the corresponding milk (AFM1 excreted in μg/day), the paired data of the samples of cattle feed and the corresponding milk samples were used. Analytical results in feed were reported as μg AFB1/kg relative to a feed with a moisture content of 12% (88% dry matter), as requested in Directive 2002/32/EC [17]. Then, the concentration of AFB1 in feed was converted into dry matter basis (μg AFB1/kg feed dry matter) by dividing by 0.88. Analytical results in milk were converted from ng AFM1/kg milk into μg AFM1/kg milk by dividing by 1000. The aflatoxin transfer rate was calculated on the basis of an average dry matter intake of 25 kg/animal/day and a milk production of 40 kg/animal/day, which were obtained from the farms investigated. Finally, in order to assess the transfer ratio of AFB1 in feed to AFM1 in milk, the following equations were used:

AFB1 ingested (μg/day) = μg AFB1/kg feed dry matter × daily dry matter intake (kg),(1)

AFM1 excreted (μg/day) = μg AFM1/kg milk × daily milk production (kg),(2)

(3) $$\mathrm{Transfer}\;\mathrm{ratio}\;(\%)\;=\;\frac{\mathrm{AFM}1\;\mathrm{excreted}\;(\mu g/\mathrm{day})}{\mathrm{AFB}1\;\mathrm{ingested}\;(\mu g/\mathrm{day})}\;\times \;100$$

### 4.7. Statistical Analysis

The identification and quantification of aflatoxins in the samples were performed using the software package OpenLAB CDC 2013 (Agilent Technologies, Barcelona, Spain). A sample was determined as positive when the result was above the limit of detection. A value of zero was assigned to the samples that presented aflatoxin concentration values lower than the LOD (limit of detection). For those samples that presented concentration values between LOD and LOQ, their numerical value was used.

Descriptive and comparative statistical analysis of the results were carried out using the IBM SPSS Statistics Base program, version 22 (Armonk, NY, USA). The data on aflatoxin B1 in feed and aflatoxin M1 in milk were not normally distributed after checking by Shapiro–Wilk test. Therefore, non-parametric tests were used for comparative statistics between factors. Firstly, Mann–Whitney U test was used so as to know if there were significant effects of feeding systems (unifeed vs. compound feed), season (spring vs. winter), and year (2015 vs. 2016) on aflatoxin levels in both feed and milk samples as well as in transfer ratios. Differences between the four sampling provinces in which the dairy farms were located were determined by Kruskal-Wallis test. All tests were carried out at a significance level of 0.05.

## Figures and Tables

**Table 1:** Summary of the occurrence of aflatoxin B1 in 60 different samples of dairy cattle feed classified by type of feeding system and by sampling season, expressed in μg/kg relative to a feed with a moisture content of 12%.

Feed Samples	*n*	% Positives ^1^	Mean ^2^ ± SD	Maximum
Unifeed rations	38	36.8%	0.39 ± 0.75	2.75
Compound feed	22	86%	0.99 ± 1.56	5.17
Spring season	35	57.1%	0.53 ± 0.89	3.29
Winter season	25	52%	0.73 ± 1.43	5.17
Total samples	60	55%	0.61 ± 1.14	5.17

^1^ AFB1 concentration above the limit of detection (LOD) of 0.03 μg/kg. ^2^ To calculate on a dry matter basis, divide the AFB1 concentration value by 0.88.

**Table 2:** Summary of the occurrence of aflatoxin M1 in 60 different samples of raw cow’s milk classified by type of feeding system and by sampling season, expressed in ng/kg.

Milk Samples	*n*	% Positives ^1^	Mean ± SD	Maximum
Cows fed unifeed rations	38	28.9%	9.0 ± 16.6	58.1
Cows fed compound feed	22	54.5%	18.8 ± 21.8	67.2
Spring milk	35	40%	15.3 ± 22.3	67.2
Winter milk	25	36%	8.8 ± 12.8	42.1
Total samples	60	38.3%	12.6 ± 19.1	67.2

^1^ AFM1 concentration above the limit of detection (LOD) of 8 ng/kg.

**Table 3:** Transfer ratio of AFM1 excreted in milk (μg/day) to AFB1 ingested with feed (μg/day) by type of feeding system and by sampling season.

Comparison	*n*	Mean AFB1 Ingested (μg/Day)	Mean AFM1 Excreted (μg/Day)	Mean Ratio AFM1/AFB1 (%)
Cows fed unifeed rations	38	11.08	0.36	2.15%
Cows fed compound feed	22	28.26	0.75	5.07%
Spring milk	35	14.94	0.61	4.17%
Winter milk	25	20.79	0.35	1.89%
Total samples	60	17.38	0.50	3.22%

**Table 4:** Ratio of AFM1 excreted in milk (μg/day) to AFB1 ingested with feed (μg/day) in 22 positive matched samples.

Sample	Feed Type	Season	AFB1 Ingested (μg/Day)	AFM1 Excreted (μg/Day)	Ratio AFM1/AFB1 (%)
1	Unifeed rations	Spring	77.25	1.20	1.55
2	Unifeed rations	Spring	15.00	0.53	3.53
3	Unifeed rations	Spring	78.25	2.19	2.8
4	Unifeed rations	Spring	62.00	1.43	2.31
5	Unifeed rations	Spring	39.00	0.88	2.26
6	Unifeed rations	Spring	43.50	1.57	3.61
7	Unifeed rations	Spring	4.50	1.62	36
8	Unifeed rations	Spring	20.00	2.32	11.6
9	Unifeed rations	Winter	34.50	0.85	2.46
10	Unifeed rations	Winter	4.00	0.62	15.5
11	Compound feed	Spring	93.50	0.80	0.86
12	Compound feed	Spring	6.75	2.01	29.78
13	Compound feed	Spring	5.50	1.09	19.82
14	Compound feed	Spring	20.75	2.64	12.72
15	Compound feed	Spring	14.00	2.69	19.21
16	Compound feed	Winter	147.00	1.69	1.15
17	Compound feed	Winter	145.00	1.10	0.76
18	Compound feed	Winter	22.00	0.96	4.36
19	Compound feed	Winter	37.50	0.74	1.97
20	Compound feed	Winter	37.50	1.18	3.15
21	Compound feed	Winter	57.50	0.89	1.55
22	Compound feed	Winter	4.75	0.78	16.42
Mean			44.08	1.35	8.79%

## Data Availability

The data presented in this study are available in article.
